# Development of a yeast internal-subunit eGFP labeling strategy and its application in subunit identification in eukaryotic group II chaperonin TRiC/CCT

**DOI:** 10.1038/s41598-017-18962-y

**Published:** 2018-02-05

**Authors:** Yunxiang Zang, Huping Wang, Zhicheng Cui, Mingliang Jin, Caixuan Liu, Wenyu Han, Yanxing Wang, Yao Cong

**Affiliations:** 1 National Center for Protein Science Shanghai, State Key Laboratory of Molecular Biology, CAS Center for Excellence in Molecular Cell Science, Shanghai Institute of Biochemistry and Cell Biology, Chinese Academy of Sciences, University of Chinese Academy of Sciences, Shanghai, China; 2 Shanghai Science Research Center, Chinese Academy of Sciences, Shanghai, China

## Abstract

Unambiguous subunit assignment in a multicomponent complex is critical for thorough understanding of the machinery and its functionality. The eukaryotic group II chaperonin TRiC/CCT folds approximately 10% of cytosolic proteins and is important for the maintenance of cellular homeostasis. TRiC consists of two rings and each ring has eight homologous but distinct subunits. Unambiguous subunit identification of a macromolecular machine such as TRiC through intermediate or low-resolution cryo-EM map remains challenging. Here we present a yeast internal-subunit eGFP labeling strategy termed YISEL, which can quickly introduce an eGFP tag in the internal position of a target subunit by homologous recombination, and the tag labeled protein can be expressed in endogenous level. Through this method, the labeling efficiency and tag-occupancy is ensured, and the inserted tag is usually less mobile compared to that fused to the terminus. It can also be used to bio-engineer other tag in the internal position of a protein in yeast. By applying our YISEL strategy and combined with cryo-EM 3D reconstruction, we unambiguously identified all the subunits in the cryo-EM map of TRiC, demonstrating the potential for broad application of this strategy in accurate and efficient subunit identification in other challenging complexes.

## Introduction

Many essential cellular processes, including transcription, translation, and protein folding and degradation, are carried out by macromolecular complexes. Precise subunit assignment in multicomponent complexes is critical for thorough understanding of the machinery and functionality of the macromolecular machine. In recent years, cryo-electron microscopy (cryo-EM) has emerged as a powerful tool for structural determination of macromolecular machines^1–8^. However, for some complexes with conformational or compositional heterogeneity, unambiguous subunit identification directly through visualization of intermediate or low-resolution negative staining (NS) or cryo-EM density map remains challenging.

Eukaryotic Group II chaperonin TRiC/CCT serves as a key protein folding nanomachine, important for the maintenance of cellular homeostasis^9–18^. TRiC is made up with two rings, each of which consists of eight homologous but distinct subunits, with their N- and C- termini buried inside the complex in the open conformation^14,19–23^. The unique subunit arrangement of TRiC underlies its functionality. Although extensive efforts have been made, unambiguous subunit identification in the cryo-EM map of TRiC is difficult due to the resolution limitation and structural similarity among its homologous subunits^19–27^. We have previously determined the locations of CCT1, CCT6, and CCT7 subunits in the open conformation cryo-EM map of TRiC in the nucleotide partially preloaded (NPP) state with the Z-shaped feature formed by an on-axis subunit pair^28^, while the locations of the other five subunits remain to be pinpointed.

Antibody or fusion tag labeling strategies have been developed for effective subunit identification in EM maps^29–33^. Still, it is usually difficult to obtain a monoclonal antibody, which has high affinity and specificity that can remain bound with target protein sustaining the NS or cryo condition, in an efficient and cost effective way. Moreover, in the fusion tag labeling strategy, eGFP, MBP, or DID tag, with certain mass and distinct shape suitable for detection in EM, has been introduced to the C- or N-terminus of the objective protein for its localization in EM map^32–36^. However, for some complexes with their terminus buried inside the complex (such as TRiC) or having certain functions, this method may induce loss-of-function or even death, and thus is not applicable. Additionally, protein termini are usually flexible and fusion tag is tethered to only one end in the target protein. Terminal labels thus often experience a large degree of freedom and adopt a variety of positions relative to the objective protein, which consequently limits accuracy in locating the position of the label relative to the subunit of interest. To overcome these limitations, internal-subunit tag labeling strategies have been developed. In a strategy termed DOLORS, it involves site-specific biotinylation of the target protein, followed by posttranslational tagging with streptavidin^37^. This method could specifically label any desired domain within a protein complex. However, the multi-step process utilized for the labeling could diminish the overall labeling efficiency and tag-occupancy of the EM images. In another recent EMIL strategy, a GFP tag was introduced in the internal position of a subunit, and the belonging complex was expressed in *Escherichia coli*^38^. This method is suitable for protein over-expression by introduction of bio-engineered plasmids, however, it is not applicable for endogenous complex expression.

Yeast is one of the optimal expression systems for endogenous expression of eukaryotic macromolecular complexes^39,40^. It is usually more difficult to introduce a tag in the internal position of a target protein than that in its terminus in yeast system. Along this line, several methods have been developed, including bioengineering proteins by heterologous promoters with exogenous vectors^40^, and modification of gene by homologous recombination. Nonetheless, the former method may encounter the over-expression problem^40,41^, while the later method will unavoidably lead to a loxP sequence that may induce unfolding or loss of function of the protein^42^ and it needs an additional sporulation process when applied to essential genes. Therefore, a more efficient and controllable internal tag insertion method is in need of.

Here we present a yeast internal-subunit eGFP labeling (YISEL) strategy, which integrates a one-step cloning and homologous recombination, allowing us to efficiently introduce a tag in the internal position of one gene in the chromosome using a PCR-based strategy^43^. Through this method, we can express the complex with internal eGFP labeled subunit in endogenous level, and the length of the linker bridging the tag and the protein is controllable. Also, through this method, the labeling efficiency and tag-occupancy is ensured, and the inserted tag is usually less mobile relative to the target protein compared to the fused tag in the terminus. Combining our YISEL strategy with cryo-EM 3D reconstruction, we were allowed to unambiguously identify all the TRiC subunits in its open NPP state cryo-EM map, demonstrating the potential broad application of this method in precise subunit identification in other macromolecular complexes.

## Results

### An efficient yeast internal-subunit eGFP labeling strategy

**Figure 1:**
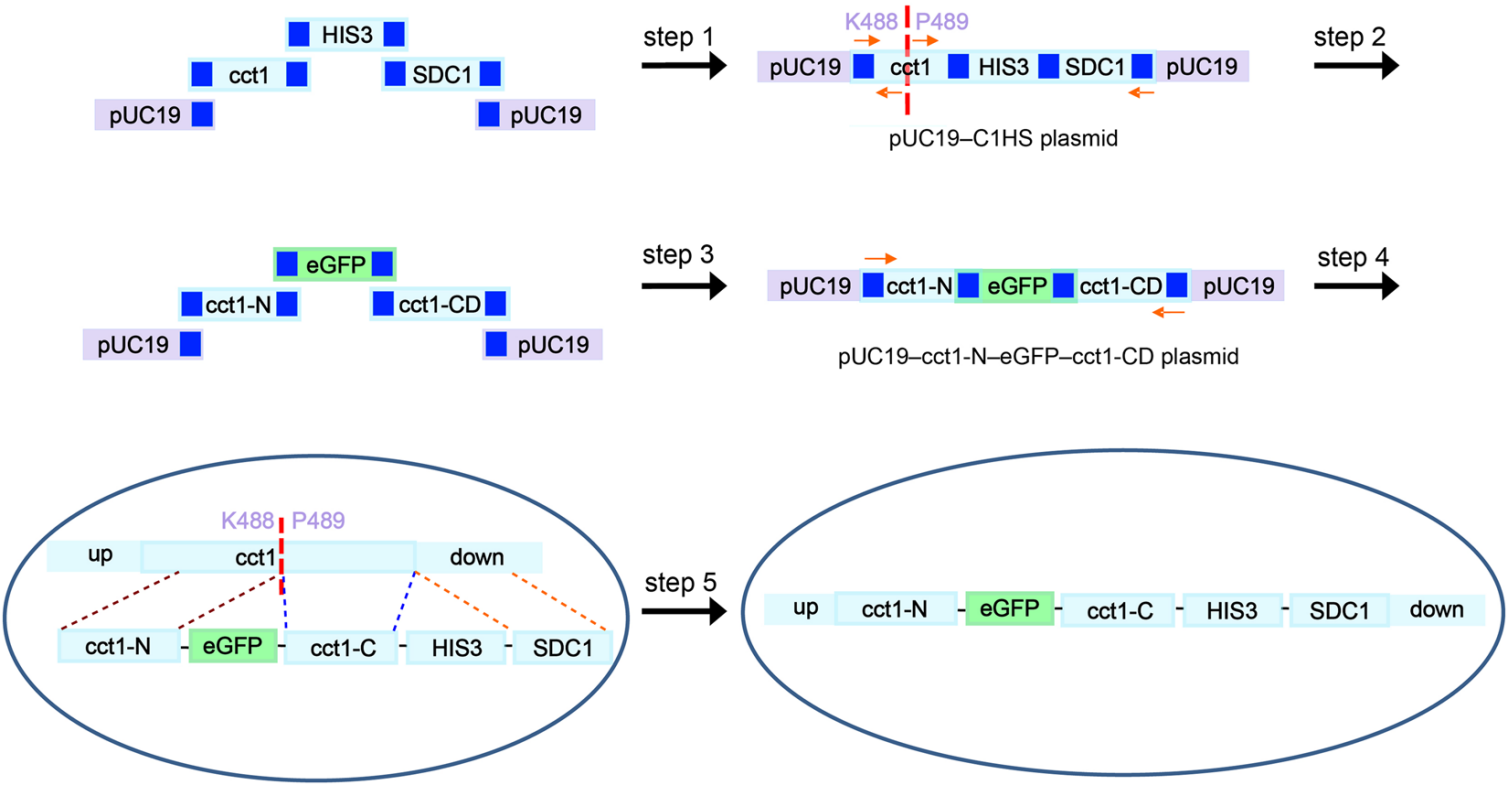
An efficient yeast internal-subunit eGFP labeling (YISEL) strategy. Taking CCT1 subunit as an example, in step 1, the PCR products of cct1 gene, HIS3 selection marker, and the sequence downstream the ORF of CCT1 subunit (~500 bp, now abbreviated as SDC1), with each fragment having a 20 bp overlapped sequence (blue square), were mixed with the linearized pUC19. The cloned DNA products were directly subjected to *E. coli* transformation for positive strain selection. In step 2, after obtaining the pUC19–cct1–HIS3–SDC1 plasmid (pUC19–C1HS), we amplified the cct1 gene upward the tag insertion site between K488 and P489 (referred as cct1-N), and the cct1 gene downward the tag insertion site together with the HIS3 selection marker and SDC1 from the pUC19–C1HS plasmid (referred as cct1-CD). In step 3, we mixed the amplified cct1-N, cct1-CD, eGFP, and the linearized pUC19, and subjected to *E. coli* transformation for positive strain selection. In step 4, we amplified the cct1-N–eGFP–cct1-CD sequence. Finally, in step 5, we transformed the purified PCR product of cct1-N–eGFP–cct1-CD to the haploid yeast, and select the positive strain.

To unambiguously locate all the subunits in the cryo-EM map of TRiC complex, we developed a yeast internal-subunit eGFP labeling (YISEL) strategy. In this strategy, we bio-engineered an eGFP tag in the middle of an individual subunit of TRiC through PCR-based homologous recombination method. We usually choose a loop region that exposed outside of the complex as the optimal tag insertion position. To obtain the full DNA fragment for yeast transformation, we adopted a one-step cloning strategy to link different fragments with vector pUC19 as the frame (Fig. 1). Since we need to perform systematic study on all the eight distinct TRiC subunits, we built a plasmid library convenient for tag insertion at any position of a specific subunit. Here every element in the plasmid library contains the complete ORF of a certain TRiC subunit, a selection marker for yeast transformation, and a ~500 bp sequence downstream the ORF of that subunit (abbreviated as SDC) (step 1 in Fig. 1, and Methods). Take CCT1 subunit as an example, its plasmid in the library was named as pUC19**–**C1HS, which was used as template for PCR in the next step. In step 2, we amplified part of the cct1 gene from the starting codon to the tag insertion site (now referred as cct1-N), and the cct1 gene downward the tag insertion site together with the HIS3 selection marker and SDC1 (now referred as cct1-CD). In step 3, to insert the eGFP tag, we mixed the amplified cct1-N, cct1-CD, and eGFP with the linearized pUC19, then the mixture was subjected to *Escherichia coli* transformation for positive strain selection. In step 4, we then amplified cct1-N**–**eGFP**–**cct1-CD based on the plasmid of pUC19**–**cct1-N**–**eGFP**–**cct1-CD. Finally, in step 5, we transformed the purified PCR product of cct1-N**–**eGFP**–**cct1-CD to the haploid yeast (BY4741 or BY4742) and select the positive strain. The positive ratio was quite high.

Utilizing this YISEL strategy, individually, we have successfully inserted an eGFP tag in six subunits of TRiC (including CCT1, CCT2, CCT4, CCT5, CCT6, and CCT7, Table S1). Using the same strategy, just by replacing the eGFP tag by a Strep-CBP-6xHis tag (abbreviated as CBP tag hereafter), we inserted a CBP tag in CCT3 for affinity purification, and all the aforementioned bioengineering was based on this TRiC CCT3-CBP strain.

### 2D image analysis is not sufficient for unambiguous subunit identification in TRiC

We then performed cryo-EM single particle analysis (SPA) on the internal-subunit eGFP labeled TRiC in the open NPP state or the both-ring closed state (in the presence of ATP-AlFx). First, we tried to examine whether reference–free 2D image analysis is sufficient to allocate tag labeled TRiC subunit in the EM structure, which represents a challenging case since the extra tag density would appear twice with one in each ring, and the homologous TRiC subunits are difficult to be distinguished in 2D class averages.

**Figure 2:**
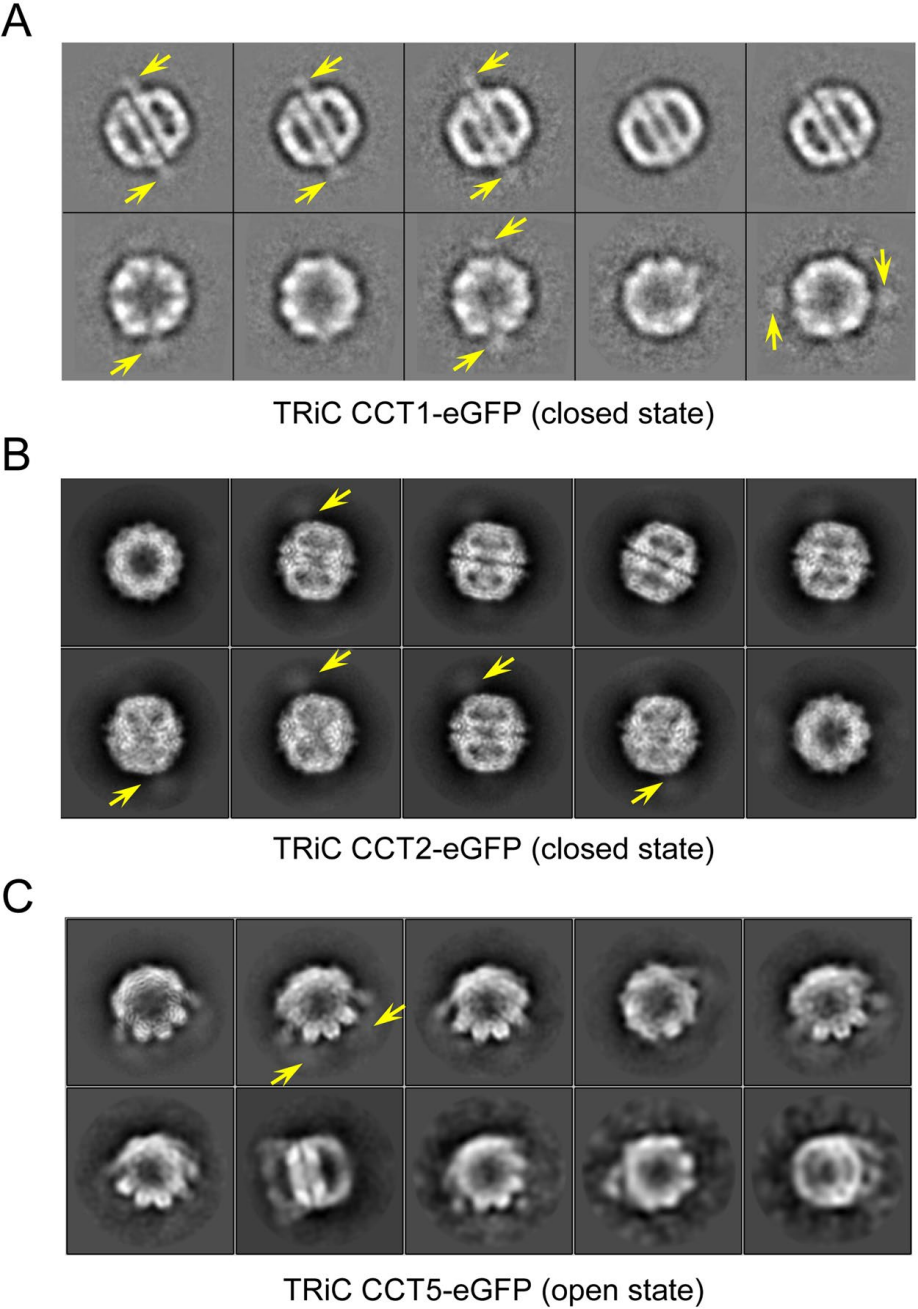
Visualization of the inserted eGFP tag in reference-free 2D class averages. (**A**) For TRiC CCT1-eGFP in the both-ring closed conformation, we can visualize the exposed extra density corresponding to the inserted eGFP tag (indicated by yellow arrow) from the reference-free 2D class averages especially in the side views. (**B**,**C**) For TRiC CCT2-eGFP in the closed state and TRiC CCT5-eGFP in the open state, although shadow density exposed outside of TRiC can be observed in the reference-free 2D class averages, the feature is rather fuzzy and it is hard to identify which subunit the tag belongs to.

Our reference-free 2D analyses reveal that TRiC CCT1-eGFP in the closed state shows the best observable tag densities (Fig. 2A). In some side-view 2D class averages, we can visualize two protruding tag densities exposed outside of TRiC in the equator, with one in each ring and located in the opposite direction (Fig. 2A), implying the relative location of the two CCT1 subunits in TRiC. However, in the top-view 2D class averages, it is difficult to simultaneously visualize two protruding tag densities and to distinguish exactly which subunit the extra density attached to. Moreover, given the overall similar conformation of the homologous TRiC subunits in the closed state, it remains hard to unambiguously identify the tag labeled subunit just through 2D analysis for a complex like TRiC.

Furthermore, for other cases, such as TRiC CCT2-eGFP in the closed state and TRiC CCT5-eGFP in the open NPP state (Fig. 2B, C), although shadow density exposed outside of TRiC can be observed in the reference-free 2D class averages, the feature is rather fuzzy and it is hard to identify which subunit the tag belongs to. Overall, we noticed that eGFP tag inserted to the equatorial domain (E-domain) of TRiC (e.g. in CCT1-eGFP) is rather stable and more distinguishable than that inserted to the intermediate domain (I-domain or apical domain (A-domain)), which is usually more dynamic especially in the open conformation of TRiC. This may also be related to the intrinsic nature of the specific loop where the eGFP tag was inserted to, that is to say for a long or dynamic loop, the inserted tag could be relative mobile. Putting together, for TRiC complex, reference-free 2D analysis alone is not sufficient to identify each of the eGFP labeled subunits. Therefore, further 3D reconstruction is necessary for unambiguous subunit identification in TRiC/CCT.

### 3D reconstruction of eGFP-labeled TRiC facilitates accurate subunit identification

**Figure 3:**
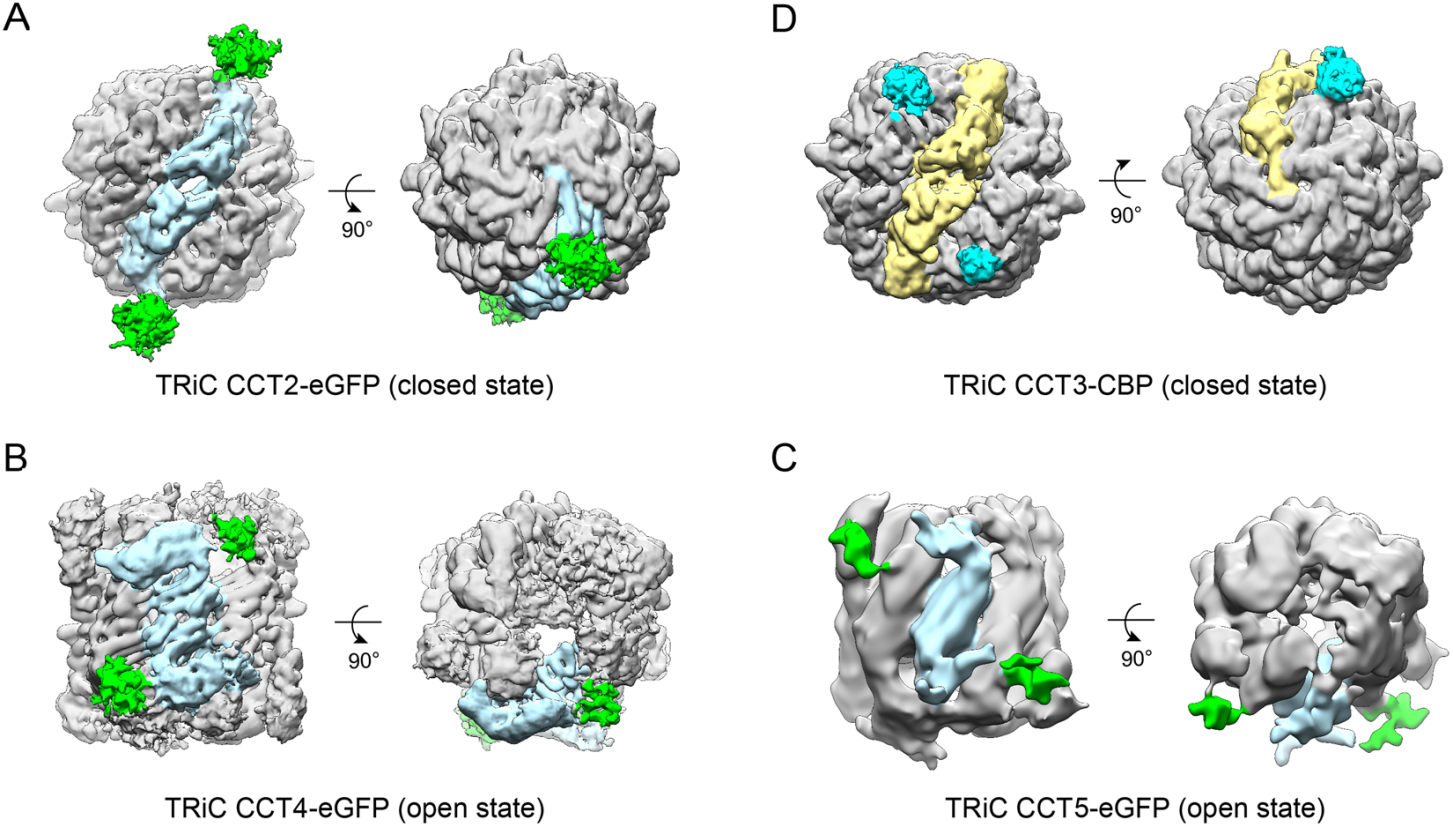
TRiC subunit identification through YISEL strategy combined with cryo-EM 3D reconstruction. (**A**) CCT2 subunit relative orientation determination in the closed state TRiC CCT2-eGFP map. Here eGFP was tagged to the A-domain of CCT2. The TRiC density is in grey, the extra density corresponding to eGFP tag in green, and the tag attached on-axis subunit in light blue. Since the tag density is usually weaker compared to that of TRiC, the map rendering threshold was lowered for better visualization of the tag density. (**B**,**C**) Direct subunit identification of CCT4 (**B**) and CCT5 (**C**) in the open NPP state TRiC CCT4-eGFP and TRiC CCT5-eGFP maps, respectively. Here eGFP was tagged to the I-domain of CCT4 or A-domain of CCT5. The adjacent Z-shaped on-axis subunit pair is in light blue. (**D**) CCT3 relative orientation determination in the closed state TRiC CCT3-CBP map. CBP was tagged to the A-I hinge region of CCT3. The on-axis subunit between the two tagged CCT3 subunits is in kaki and the extra density corresponding to CBP tag is in cyan.

**Figure 4:**
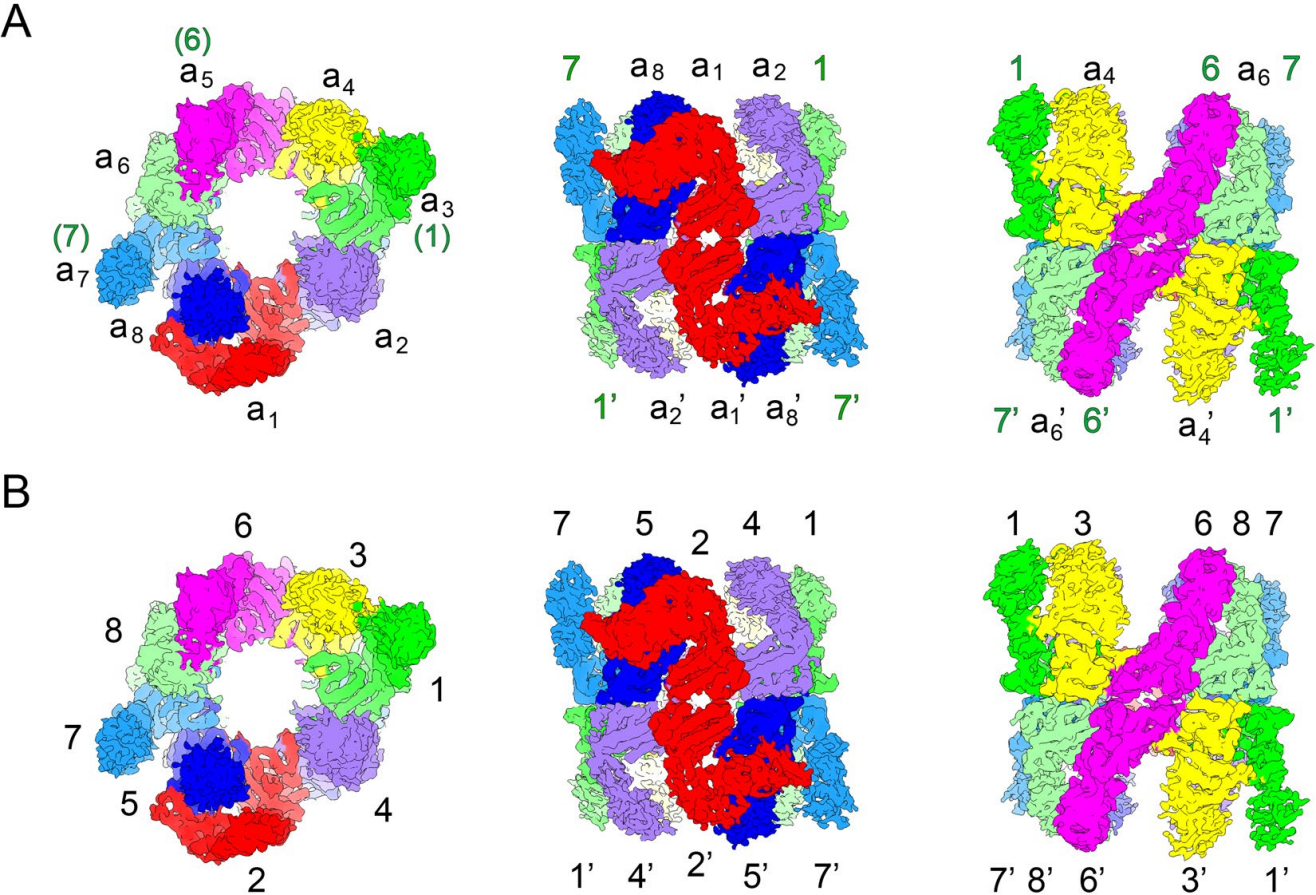
TRiC subunit assignment in the open NPP state cryo-EM map. (**A**) In the open NPP state cryo-EM map of TRiC (EMD-9540)^28^, the subunits are numbered sequentially, starting from one of the on-axis subunits with the bent feature. Different subunits are in different colors. The previously determined subunits CCT1, CCT6, and CCT7 are also labeled in green. (**B**) Subunit assignment in the open NPP state TRiC map, determined by applying the YISEL strategy. Different views of the map were shown.

To unambiguously assign all the subunits of TRiC in the density map, we performed cryo-EM 3D reconstructions on the tag labeled TRiC complexes, including TRiC CCT2-eGFP, TRiC CCT4-eGFP, TRiC CCT5-eGFP, and TRiC CCT3-CBP, in open or closed conformation (Fig. 3, S1, Tables S1, S2). The TRiC CCT2-eGFP map in the closed state reveals two subunits with extra density (one in each ring) stand on top of each other (Fig. 3A), indicating that CCT2 is one of the two on-axis subunits (a_1_ and a_5_) located on the two-fold symmetrical axis (Fig. 4A). We have previously assigned CCT1, CCT7, and CCT6 subunits in the open NPP state cryo-EM map of TRiC^28^, and depicted that CCT6 is the on-axis subunit a_5_ without the bent feature in this state (Fig. 4A). Therefore, the other on-axis subunit a_1_ with the unique bent feature in NPP state should be CCT2 (Fig. 4). Moreover, we resolved the cryo-EM maps of TRiC CCT4-eGFP and TRiC CCT5-eGFP in the open NPP state (Fig. 3B,C). Overall, these two maps are in the same conformation as that of the wild-type NPP-TRiC map with the Z-shaped feature^28^ (Fig. 4A), which allows us to correlate these two maps with the wild-type NPP-TRiC map. These analyses reveal that CCT5 and CCT4 locate immediately besides the bent CCT2 subunit with one in each side. Their relative location to CCT2 suggests that subunit a_2_ should be CCT4 and subunit a_8_ should be CCT5 (Fig. 4).

Here we have assigned CCT2, CCT4, and CCT5 subunits in the cryo-EM map of NPP-TRiC; combining with previously assigned CCT1, CCT7, and CCT6 subunits^28^ (Table. S1), there are only two remaining subunits (CCT3 and CCT8) need to be further identified in the map. Accordingly, there are two unassigned subunits, a_4_ and a_6_, located next to CCT6 in each side (Fig. 4A). For CCT3 subunit, we had engineered a CBP tag in the hinge region between its A- and I-domains (abbreviated as A-I hinge region) for affinity purification, therefore no additional tag was inserted in this subunit. In the closed state TRiC CCT3-CBP cryo-EM map, we can clearly observe two extra densities correlated to the CBP tag (Fig. 3D). The relative location of the two tag densities depicts that CCT3 should reside immediate beside one of the on-axis subunit. Since we have determined that one of the on-axis subunit CCT2 is surrounded by CCT4 and CCT5 (Fig. 3B,C, and Fig. 4B), the remaining on-axis subunit neighboring to CCT3 should be CCT6. Putting together, subunit a_4_ should be CCT3 (Fig. 4). Moreover, for CCT8, we have tried to insert an eGFP tag in different locations of this subunit (including Q375, T151, and D493), but did not succeed. Still, since we have identified all the other seven subunits in the cryo-EM map of NPP-TRiC, the only remaining subunit a_6_ next to CCT6 should be CCT8 (Fig. 4). Until now, we have unambiguously assigned all the TRiC subunits in its open NPP state cryo-EM map for the first time by applying the YISEL strategy and direct visualization of the tag labeled cryo-EM maps. Overall, the TRiC subunit ordering determined here is in line with previous XL-MS result^24,26^; still, we would point out that only through this YISEL strategy, we were allowed to precisely assign these homologous TRiC subunits in the cryo-EM map that not yet having the atomic resolution structural details.

## Discussion

In this study, we developed an efficient yeast internal-subunit eGFP labeling strategy termed YISEL, which, without inducing any additional loxP sequence, allows us to express yeast complexes in the endogenous level with an inserted eGFP tag in the internal position of the target protein. In the produced complex, the labeling efficiency and tag-occupancy is ensured, and the inserted tag is usually less mobile compared to the fused tag in the terminus. Eukaryotic chaperonin TRiC/CCT remains a challenging system for unambiguous subunit identification due to the homologous nature of its eight subunits and resolution limitation of available structures. By applying this YISEL strategy, we have successfully assigned all the TRiC subunits in the cryo-EM density map. Our results demonstrate that the YISEL strategy could serve as a robust tool for accurate and efficient subunit identification in other challenging macromolecular complexes with their map at intermediate or low resolution.

In terms of the stability of the inserted tag, in the classical method, the tag is usually fused to the terminus of a subunit with only one end attached to the target protein, consequently the tag is usually mobile and may swinging away from the target subunit. This makes it difficult to accurately identify the objective subunit through 2D or 3D EM analysis. Moreover, in some challenging cases, the terminus of the objective subunit may be buried inside the complex, or fusion a tag in the terminus may be lethal for the complex. However, in our YISEL strategy, since both ends of the eGFP tag are inserted into the middle of a subunit, the tag usually appears more stable and stays relative close to its target subunit, beneficial for accurate subunit identification.

Several key factors need to be considered when applying this YISEL strategy, including the insertion location of the tag, the length of the loop hosting the inserted tag, the length of the inker, as well as the size of the tag. We found that, in TRiC, the tag inserted in the more stable E-domain is easier to be identified than that in I-domain or A-domain, and in general the exposed loop with suitable length and not too flexible is more ideal for eGFP tag insertion. In addition, insertion of eGFP with shorter or no linker (e.g. no linker in the CCT6-eGFP case) usually leads to a more stable tag that is easier to be identified in the 3D map (Table S1)^28^, but may hinder the growth of the strain. Moreover, utilizing the same recombinant method, we also tried to insert a larger MBP tag (~42 kDa) in CCT2 subunit in the same position where eGFP was inserted (Table S1). We found that MBP tag can be inserted but must with the GSGSG linker in both ends, and this strain has a slower growth rate compared to that of the CCT2-eGFP strain in the same condition (data not shown), suggesting that the size of the tag and the length of the linker matter for the successful internal-subunit tag insertion.

## Methods

### Molecular biology

For convenient tag insertion at any position in all the TRiC subunits, we built a plasmid library (Fig. 1 and Table S1). Each plasmid in the library contains the complete ORF of a certain TRiC subunit, a selection marker for yeast transformation, and a ~500 bp downstream the ORF of that TRiC subunit (abbreviated as SDCn, where n refers to CCTn subunit). Such plasmid was named as pUC19-CnHS. The plasmid used in this study was constructed with the one-step cloning kit (vazyme). Take TRiC subunit CCT1 as an example, to construct the pUC19-C1HS plasmid in the library, the cloning vector, pUC19, was linearized by digestion with restriction endonuclease (BamHI and HindIII) before applying to recombination reaction. The overlapped gene sequence guiding the recombination reaction was prepared by PCR amplification using primers with homologous recombination sequences added to their 5′ ends. The PCR products of intact cct1 ORF gene, HIS3 selection marker, and the sequence of SDC1, with each fragment having a 20 bp overlapped sequence (blue square in Fig. 1), were mixed with the linearized pUC19 and proceeded for recombination reaction. The recombination reaction was set up at 37 °C for 30 min to assemble gene fragments to a complete plasmid. The reaction product was directly transformed to *Escherichia coli* DH5α competent cells for positive strain selection. After gene sequencing, we obtained the pUC19-cct1-HIS3-SDC1 plasmid (pUC19-C1HS) as one of the plasmids in the library (Fig. 1, step 1).

Subsequently, the eGFP tag was thus inserted into the targeting internal position of a subunit also using the PCR-based strategy, with pUC19-CnHS in the library as template (Fig. 1, step 2). We still take CCT1 subunit as an example, three different gene fragments (including cct1-N, eGFP, and cct1-CD) were mixed with linearized pUC19 and underwent recombination reaction. After transformation and positive strain selection, a plasmid with eGFP inserted into target position was constructed (Fig. 1, step 3), which was named as pUC19–cct1-N–eGFP–cct1-CD plasmid. This plasmid was the template for obtaining the whole sequence including eGFP for subsequent yeast transformation (Fig. 1, step 4). Finally, through yeast homologous recombination method, an eGFP tag was inserted into the target location in the yeast strain containing CBP tag in CCT3 for affinity purification (Fig. 1, step 5). The location of CBP tag insertion in CCT3 was adopted from a previous study, but no details was reported on their insertion strategy^44^.

For CCT8, we have tried to insert an eGFP tag in distinct locations of this subunit (including Q375, T151, and D493), but did not succeed. For each location, we have obtained the PCR fragment that can be confirmed by sequencing, but failed to obtain the positive strain in yeast transformation. Recently, it has been reported that CCT8 subunit might be a key modulator of TRiC/CCT assembly^45^. Therefore, modification of CCT8 subunit such as insertion of an eGFP tag might be lethal for yeast.

### Protein purification

Yeast TRiC with inserted tag was purified according to a published protocol^28,44^. The supernatant of yeast lysate was incubated with calmodulin resin (GE Healthcare) overnight at 4 °C. Elution of TRiC was achieved by using elution buffer with 2 mM EGTA. The pooled elute containing TRiC was concentrated with a Millipore Ultrafree centrifugal filter device (100 kDa cutoff).

### Cryo-EM sample preparation and data collection

To prepare the open NPP state TRiC sample, including TRiC CCT4-eGFP and TRiC CCT5-eGFP, 2.1 µl sample was applied to a glow-discharged holey carbon grid (Quantifoil, R1.2/1.3, 200 mesh). The grid was blotted and then plunged into liquid ethane cooled by liquid nitrogen utilizing Vitrobot Mark IV (FEI). To address the preferred orientation problem usually associated with group II chaperonins, the grid was pretreated with polylysine^7,28^. Moreover, to prepare the both-ring closed TRiC, purified TRiC CCT2-eGFP or TRiC CCT3-CBP sample was incubated in the presence of 1 mM ATP, 5 mM MgCl_2_, 5 mM Al(NO_3_)_3_, and 30 mM NaF for 1 h at 30 °C before freezing^19^. The same sample vitrification process used for open-state NPP-TRiC was followed for TRiC in the closed ATP–AlFx state.

Data collection was performed on a Titan Krios electron microscope (FEI) operated at an acceleration voltage of 300 kV, with a nominal magnification of 18,000 × . The images were recorded on a K2 Summit direct electron detector (Gatan) operated in counting mode yielding a pixel size of 1.3 Å (Table S2), with a defocus range of −1.0 to −3.0 µm. Each frame was exposed for 0.2 s, with an accumulation time of 7.6 s for each movie, thus leading to a total accumulated dose of 38 e^−^/Å^2^ on the specimen. All of the imaging conditions are listed in Table S2.

### Cryo-EM 3D reconstruction

Image processing and 3D reconstruction processes are listed in Table S2. Motion corrections were performed with Motioncorr^46^ before further image processing. CTF parameters of each image were determined with CTFFIND3^47^. Particles were picked automatically with Relion1.3^48^, and were further cleaned up through 2D classification. Reference-free 2D image analysis was performed in EMAN2^49,50^ or Relion1.3. As for the initial model, we used a previous asymmetric bovine apo-TRiC structure^20^, but was eight-fold-symmetrized (C8 symmetry, but no symmetry was imposed between the two rings) and then low-pass-filtered to 60 Å resolution. No symmetry was imposed in our 3D classification process. Autorefine was performed on the major class. The resolution estimation was based on the gold-standard Fourier shell correlation (FSC) 0.143 criterion. The resolution of the cryo-EM maps of TRiC CCT2-eGFP, TRiC CCT3-CBP, TRiC CCT4-eGFP, and TRiC CCT5-eGFP was estimated at 7.6 Å, 6.9 Å, 7.9 Å, and 23.8 Å, respectively (Table S2). Here the resolution of the TRiC CCT5-eGFP map is worse than that of the others (Fig. 3). Compared to the other samples, the TRiC CCT5-eGFP sample is prone to be broken and the particle number of this dataset is less than the others (Table S2). The fragile nature of the sample might be related to the insertion location of eGFP tag in CCT5 subunit, which is in the A-domain but adjacent to the I-domain, where the bent CCT2 subunit attached to in the open NPP state (Fig. 4B). It is possible that the inserted eGFP tag in CCT5 might affect the attachment of CCT2 to CCT5, making TRiC CCT5-eGFP less stable and prone to be broken. Even though the resolution is relative low, we were still allowed to identify the eGFP labeled CCT5 subunit in the open state map of TRiC CCT5-eGFP (Fig. 3C). The maps were all rendered using UCSF Chimera^51^.

### Data availability statement

All of the data that support the findings of this study are available from the corresponding author upon reasonable request.

## Electronic supplementary material

Supplementary information
