# Supplementary material for: Development of a yeast internal-subunit eGFP labeling strategy and its application in subunit identification in eukaryotic group II chaperonin TRiC/CCT
Source: Sci Rep. 2018 Feb 5;8:2374. doi: 10.1038/s41598-017-18962-y (PMC5799240; doi:10.1038/s41598-017-18962-y)
Supplement: Supplementary file 1 — Supplementary information [file 41598_2017_18962_MOESM1_ESM.pdf]

Development of a yeast internal-subunit eGFP labeling strategy and its application in subunit identification in eukaryotic group II chaperonin TRiC/CCT

Yunxiang Zang[1, #], Huping Wang[1, #], Zhicheng Cui[1], Mingliang Jin[1], Caixuan Liu[1], Wenyu Han[1], Yanxing Wang[1, 2], Yao Cong[1, 2, *]

[1] National Center for Protein Science Shanghai, State Key Laboratory of Molecular Biology, CAS Center for Excellence in Molecular Cell Science, Shanghai Institute of Biochemistry and Cell Biology, Chinese Academy of Sciences, University of Chinese Academy of Sciences, Shanghai, China;

[2] Shanghai Science Research Center, Chinese Academy of Sciences, Shanghai, China;

[#]These authors contributed equally to this work.

*Correspondence should be addressed to Y.C. (cong@sibcb.ac.cn).

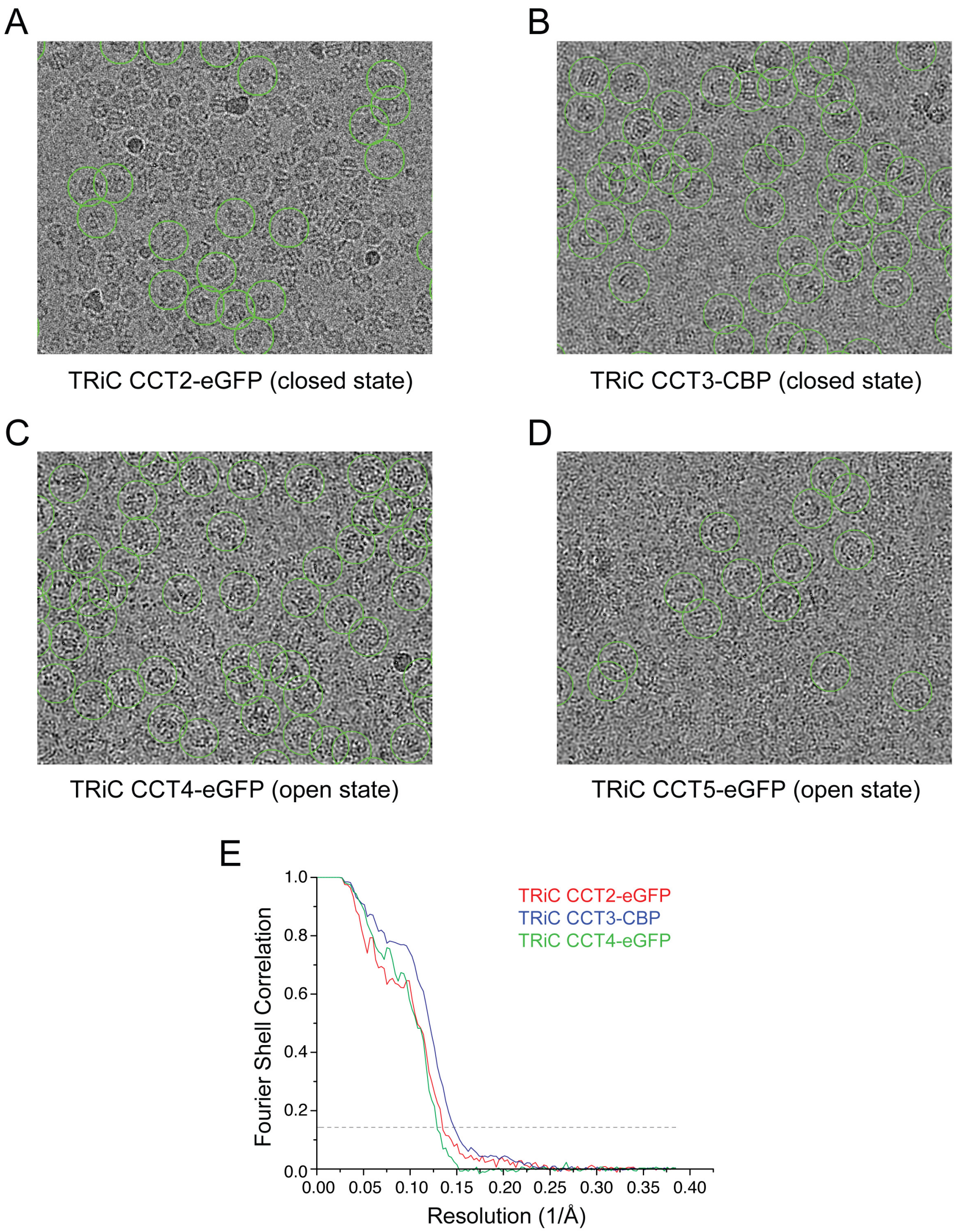

Figure S1 Representative cryo-EM images and resolution estimation. (A, B) TRiC CCT2-eGFP and TRiC CCT3-CBP in the closed state (in the presence of ATP-AlFx). (C, D) TRiC CCT4-eGFP and TRiC CCT5-eGFP in the open NPP state. (E) Resolution estimation of the TRiC CCT2-eGFP (red curve), TRiC CCT3-CBP (blue curve), and TRiC CCT4-eGFP (green curve) maps according to the gold-standard FSC criterion of 0.143.

Table S1 Insertion location of eGFP tag in TRiC subunits, linker information, and cryo-EM 3D reconstructions performed.

	Insertion site	domain	linker	open** state	closed** state
CCT1	K488|P489	E	GSGSG, GSGSG	✔	✔
CCT2	K334|C335	A	GSGSG, GSGSG		✔
CCT3*	P374|K375	A-I	n/a		✔
CCT4	N185|S186	I	GSGSG, GSGSG	✔	✔
CCT5	G263|S264	A	GSGSG, GSGSG	✔	
CCT6	P373|K374	A-I	n/a	✔	✔
CCT7	E150|K151	I-E	GSGSG, GSGSG	✔	
CCT8					

* For CCT3, a CBP tag (containing Strep, CBP and 6xHis) was inserted instead of eGFP tag.

** 3D reconstructions performed in the current study are shown in red, and in previous study in green[28], through which we determined the locations of subunits CCT1, CCT6, and CCT7 in the map.

Table S2 Statistics of cryo-EM data collection, processing, and structure refinement.

	TRiC CCT2-eGFP closed state	TRiC CCT3-CBP closed state	TRiC CCT4-eGFP open state	TRiC CCT5-eGFP open state
Detector	K2 Summit	K2 Summit	K2 Summit	K2 Summit
Defocus range (μm)	-1.5~-3.5	-1.5~-3.5	-1.5~-3.0	-1.5~-3.0
Pixel size (Å)	1.3	1.3	1.3	1.3
Electron dose ($e^-/Å^2$)	25	38	38	38
Particles refined	34,300	42,097	45,451	15,240
Final Resolution (Å)	7.6	6.9	7.9	23.8
